# Case Report: A rare case of non-small cell lung cancer with STRN-ALK fusion in a patient in very poor condition treated with first-line ensartinib

**DOI:** 10.3389/fonc.2023.1235679

**Published:** 2023-09-22

**Authors:** Guo-qiang Song, Yi-zhong Li, Weiliang Kong, Guo-qiang Hu

**Affiliations:** ^1^ Department of Respiratory, Changxing County Hospital of Traditional Chinese Medicine, Huzhou, China; ^2^ Department of Respiratory, Changxing County Jiapu Town Health Center, Huzhou, China; ^3^ Department of Pathology, Changxing County Hospital of Traditional Chinese Medicine, Huzhou, China

**Keywords:** STRN-ALK, NSCLC, ensartinib, case report, TKIs

## Abstract

Several cases of STRN-ALK fusion have been reported, and some anaplastic lymphoma kinase (ALK) inhibitors have been shown to be effective for treatment. Nevertheless, no cases of COVID-19 leading to heart failure and respiratory failure have been reported in people older than 70 years treated with ALK inhibitors. The present case report describes a 70-year-old patient with usual chronic obstructive pulmonary disease, diabetes, depression, and carotid plaque disease. Next-generation sequencing of tissue obtained by puncture biopsy revealed a STRN-ALK mutation accompanied by a TP53 mutation. The patient was treated with ensartinib and developed COVID-19 leading to heart failure and respiratory failure; nevertheless, he had a good clinical outcome and exhibited high treatment tolerability.

## Introduction

The overall incidence of non-small cell lung cancer (NSCLC) is high in patients of all ages (1).Approximately 1 to 8 million cases of lung cancer are newly diagnosed each year, and 1 to 6 million deaths are related to this disease. Lung cancer is the leading cause of cancer-related death globally (2). China has the highest lung cancer-related mortality rate, with NSCLC accounting for the majority of cases (3). In 3% to 7% of patients with NSCLC, chromosomal rearrangements involving anaplastic lymphoma kinase (ALK) have been identified as oncogenic factors (4, 5). The clinical outcomes and prognosis of patients treated with tyrosine kinase inhibitors (TKIs) are better than those of patients treated with conventional chemotherapy drugs (6).

With the recent development of diagnostic methods, next-generation sequencing (NGS) to detect ALK fusions has become more helpful in driving treatment decisions (7). Additionally, some rare ALK fusions have been detected in the clinical setting (8). This report describes a patient with STRN-ALK fusion who could not tolerate surgery and received ensartinib as first-line treatment. He thereafter developed COVID-19 (omicron variant) but nevertheless demonstrated good tolerability and clinical efficacy.

## Case report

A 70-year-old man was admitted to our hospital because of an acute attack of chronic obstructive pulmonary disease (COPD) (recurrent cough and sputum for more than 5 years, aggravated by recurrence for 1 week). The patient had type 2 diabetes mellitusinternal carotid artery plaque and history of depression. The patient had smoked an average of 30 cigarettes a day for 50 years. Physical examination only found mild edema of both lower extremities.

**Figure 1:**
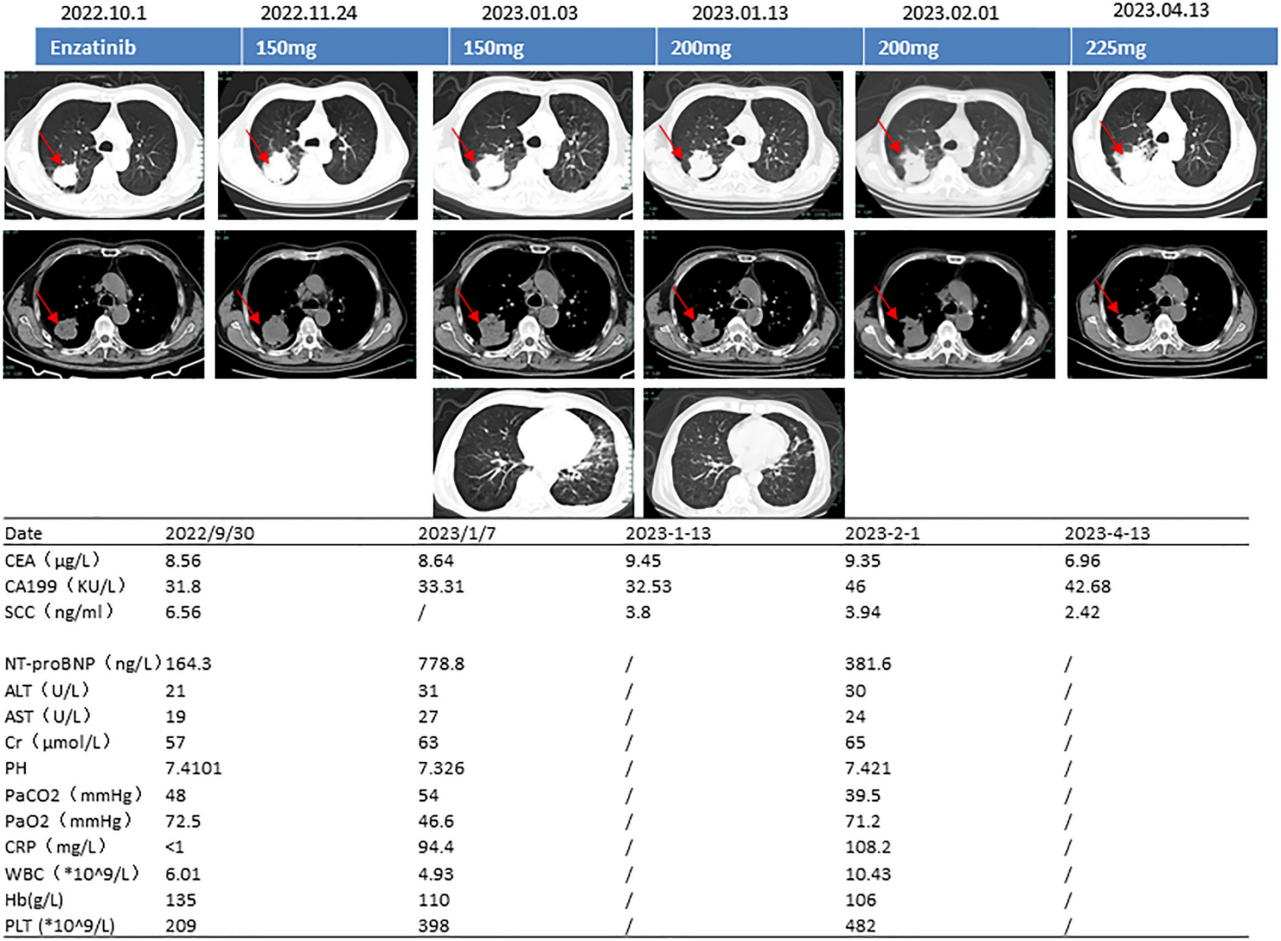
Computed tomography images, ensartinib dose, and blood test results. CEA, carcinoembryonic antigen; CA19-9, carbohydrate antigen 19-9; SCC, squamous epithelial cell carcinoma antigen; NT-proBNP, N-terminal brain natriuretic peptide precursor; ALT, alanine transaminase; AST, aspartate transaminase; pH, potential of hydrogen; PaCO_2_, partial pressure of carbon dioxide; PaO_2_, partial pressure of oxygen; CRP, C-reactive protein; WBC, white blood cell count; Hb, hemoglobin; PLT, platelet count.

Computed tomography (CT) of his chest on 29 September 2022 showed a mass (4.2 × 3.8 cm) in his right upper lung with cavity formation. Emphysema and microscopic nodules were present in the left upper lung and right lower lung (**Figure 1**).

Routine blood testing on 29 September 2022 showed the following results: C-reactive protein, <1.0 mg/L; white blood cell count, 6.01 × 10^9^/L; hemoglobin, 135 g/L; and platelet count, 209 × 10^9^/L. Arterial blood gas analysis indicated a pH of 7.41, partial pressure of carbon dioxide (PaCO_2_) of 48.0 mmHg, and partial pressure of oxygen (PaO_2_) of 72.5 mmHg. The concentration of glycosylated hemoglobin was 9.70%. The D-dimer concentration was normal. The concentration of N-terminal natriuretic peptide precursor in his brain was 164.30 ng/L (reference range, 0–125 ng/L). The concentration of carcinoembryonic antigen (CEA) was 8.56 μg/L (reference range, 0–5 μg/L), and the concentration of squamous epithelial cell carcinoma antigen (SCC) was 6.560 ng/mL (reference range, 0–3 μg/L).

**Figure 2:**
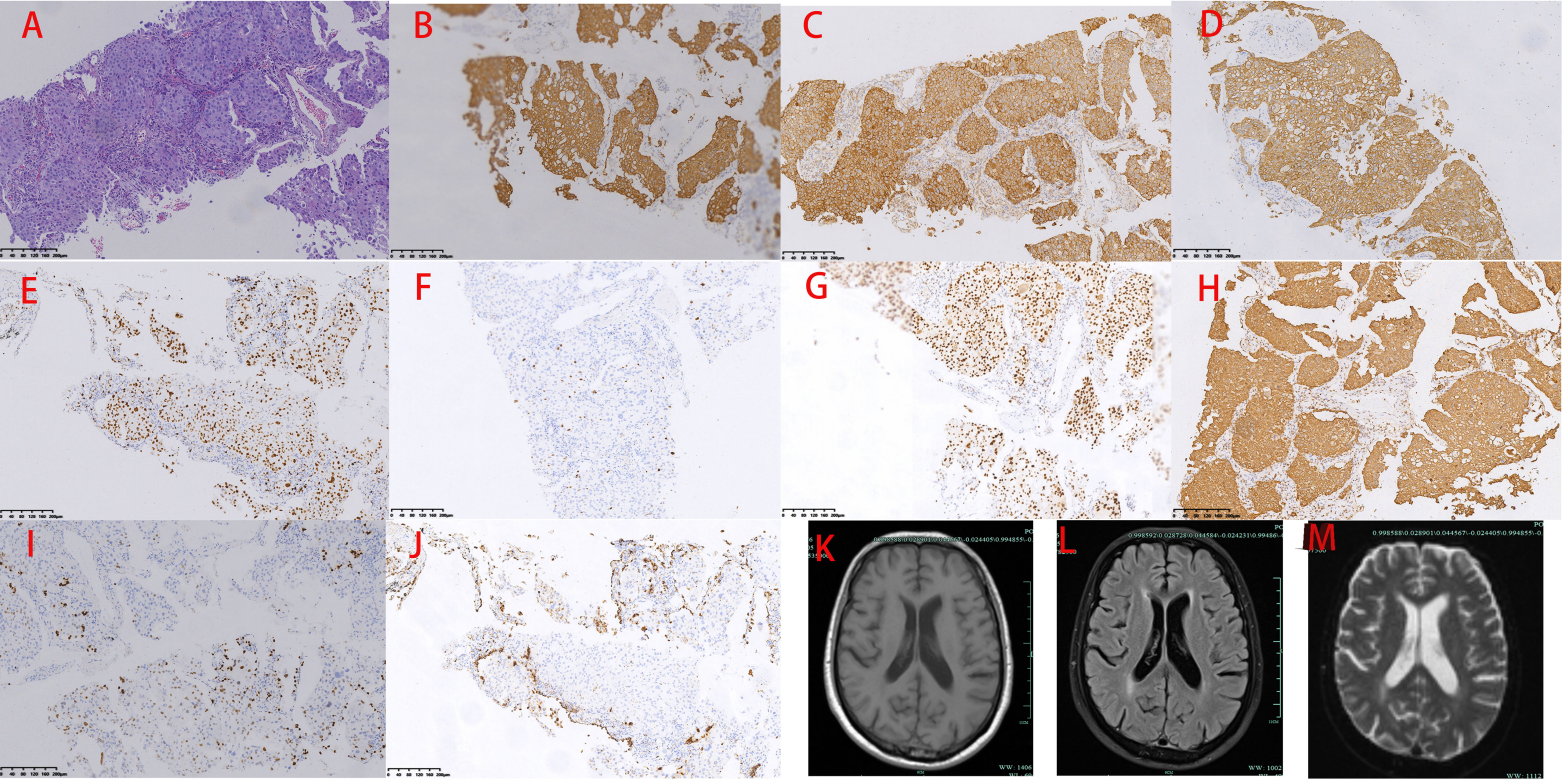
Pathology and magnetic resonance imaging. **(A)** Hematoxylin–eosin. **(B)** Cytokeratin 5/6 (+). **(C)** Epidermal growth factor receptor (+). **(D)** Cytokeratin 7 (+). **(E)** Ki67 (+, 60%). **(F)** p40 (few+). **(G)** p53 (+++). **(H)** Pan-cytokeratin (+). **(I)** Thyroid transcription factor-1 (few +). **(J)** Napsin A (few +). **(K)** T1-weighted imaging. **(L)** Fluid-attenuated inversion recovery. **(M)** T2-weighted imaging.

Pulmonary function testing revealed very severe mixed ventilation dysfunction, severely reduced diffusion function, and a negative bronchial diastolic test (FEV1 0.87L/S, FEV1% 32.1, FEV1/FVC 41.63%). CT-guided pulmonary puncture biopsy was performed on 3 October 2022. Enhanced magnetic resonance imaging on 5 October 2022 showed no tumor metastasis in brain (**Figures 2K–M**). 10 October 2022 (combination of hematoxylin–eosin staining and immunohistochemistry) showed non-small cell carcinoma. The immunohistochemical results were as follows: carcinoembryonic antigen (−), cytokeratin 5/6 (+), epidermal growth factor receptor (EGFR) (membrane +), cytokeratin 7 (+), Ki-67 (+, 60%), p40 (few +), p53 (3+), pan-cytokeratin (+), thyroid transcription factor-1 (few +), napsin A (few +), cytokeratin 20 (−), and GATA-3 (+) (**Figures 2A–J**). On 11 October 2022, kidney, ureter, and bladder ultrasound showed no abnormal findings. The patient had poor lung function and could not tolerate surgery, and he refused other tests and treatments. Therefore, we recommended pulmonary function exercise. A repeat CT examination of his chest 1.5 months later showed that the lung mass had grown (5.9 × 5.4 cm) and that the sub-rhomboid lymph nodes had become enlarged (**Figure 1**). The patient’s Eastern Cooperative Oncology Group performance status (PS) score was 2.

The patient underwent next-generation sequencing, which showed STRN-ALK (STRN_exon3-ALK_exon20) fusion mutation (1.70%) and TP53 NM_000546 exon5 c.473G>T p.R158L (14.10%).

Considering the patient’s underlying concomitant diseases and poor PS score, we prescribed ensartinib at a starting dosage of 150 mg/day. One month later, repeat CT of his chest showed that the tumor was smaller and that the treatment had resulted in stable disease. The patient experienced no intolerance even after increasing the dosage to 200 mg/day. Two months later, however, he became infected with the omicron variant of SARS-CoV-2, resulting in type 1 respiratory failure and heart failure (pH, 7.326; PaCO_2_, 54 mmHg; PaO_2_, 46.6 mmHg; NT-proBNP, 778.8 ng/L). We carried out targeted treatment and continued the ensartinib. At follow-up on 13 April 2023, the tumor was stable but had slightly increased in size (5.3 × 5.5 cm) (**Figure 1**). We thereafter recommended increasing the dosage of ensartinib to 225 mg/day, and no additional adverse effects were noted at the subsequent telephone follow-up.

## Discussion

ALK is a member of the insulin receptor family of receptor tyrosine kinases, which are expressed in different types of cancers (9). In normal cells, ALK sits on cell membranes and interacts with growth factors. EML4 is the most common ALK rearrangement in NSCLC. With advances in clinical genetic testing technology, more genes that are coexpressed with ALK have been identified (6, 10, 11). STRN-ALK fusion is a rare type of ALK rearrangement in NSCLC (12).

The STRN gene is located on chromosome 2 and encodes a protein with a coiled-coil domain that leads to constitutive activation of ALK *via* dimerization (8). Treatment options for patients who have NSCLC with rare ALK fusion often rely on past case reports. Many different ALK inhibitors have been reported to be effective for treating STRN-ALK, including crizotinib, ceritinib, ensartinib, and alectinib (8, 12–14).

We retrieved 11 reports describing cases of STRN-ALK in PubMed (**Table 1**). At the time of identification of the STRN-ALK mutation, gefitinib had previously been used in two cases, crizotinib in two cases, and alectinib in two cases. Of all 11 patients, 9 were male, at least 7 were Chinese, and 8 were nonsmokers. Five patients had right-sided lesions, five had left-sided lesions, and one was not described. Adenocarcinoma was present in 10 patients, and the pathologic type was not described in 1 patient. Pulmonary embolism occurred in two patients, and bone metastases were present in at least six. Most of the patients underwent NGS, mostly using tissue for testing. The STRN-ALK mutation rate ranged from 0.04% to 27.11% (15–18).

**Table 1:** Details of all published cases of non-small cell lung carcinoma with STRN-ALK fusion.

NO.	Author	Publised Year	Sex	Age	Race	Smoker	Test method	Sample	Side	Pathologic diagnosis	TNM	PTE	Bone metastases.	Before find STAN-ALK used TKIs	STAN-ALK frequency	First line	Time of Response/Sustained response (Months)	Second line	Time of Response/Sustained response (Months)	Third line
1	Yang Y	2017	Male	59	Chinese	No	NGS	Surgical sample	Right	Adenocarcinoma	Postoperative metastases	No	Yes	No	1%(first test) to 9%(second test)	Crizotinib	1/6	/		
2	Nakanishi Y	2017	Male	51	No declare	No	RNAseq	Biopsy tissue	Left	Adenocarcinoma	IV	Yes	No	Crizotinib	No declare	Aectinib	No respoes	Docetaxel	No respoes	/
3	Ren H	2019	Female	52	Caucasian,	No	RNAseq	Biopsy tissue	Right	Adenocarcinoma	IV	No	Yes	Crizotinib	No declare	Ceritinib	No delcare/26	/		
4	Zhou C	2019	Male	43	Chinese	Yes	NGS	Biopsy tissue	Right	Adenocarcinoma	IV	No	Yes	Gefitinib	No declare	Gefitinib plus Crizotinib	No delcare/6	/		
5	Su C	2020	Male	64	Chinese	No	NGS	Blood cell	Left	Adenocarcinoma	IVB	No	Yes	No	STRN 8.7% ,ALK 15.9%	Alectinib	0.5/17	/		
6	Nagasaka M	2020	Male	66	No declare	No	NGS	No declare	No delare	Adenocarcinoma	IV	Yes	No	Alectinib	No declare	Docetaxel	No delcare	/		
7	Sun K	2021	Male	65	Chinese	Yes	NGS	Biopsy tissue	Left	Adenocarcinoma	IVA	No	Yes	No	0.04%	Alectinib(1.5m)	1/5	Crizotinib	1/4	Chemotherapy
8	Zeng H	2021	Female	29	Chinese	No	NGS	Biopsy tissue	Right	Solid-predominant adenocarcinoma	IVB	No	Yes	No	27.11%	Alectinib	1/7	/		
9	Zeng Q	2021	Male	38	No declare	No	NGS	patient’s plasma	Left	No declare	IV	No	Yes	Gefitinib	0.70%	Crizotinib and osimertinib.	2/5	/		
10	Li M	2021	Male	42	Chinese	No	NGS	Biopsy tissue	Right	Adenocarcinoma	Postoperative metastases	No	No	Aectinib	25.90%	Crizotinib	1/11	/		
11	Zhang L	2022	Male	67	Chinese	Yes	NGS	Biopsy tissue	Left	Adenocarcinoma	IVa	No	No	No	24%	Crizotinib(1m,18m)	2/18.5	Ensartinib	No delcare/13	/

NGS, next-generation sequencing; RNAseq, RNA sequencing; PTE, pulmonary thromboembolism; TP53, tumor protein p53; STRN, striatin; ALK, anaplastic lymphoma kinase; TKIs, tyrosine kinase inhibitors.

Various other mutations were present in combination with STRN-ALK, including EGFR exon 19 deletion, TP53, and PIK3CA. First-line crizotinib was used in 5 of the 11 patients, with maintenance of efficacy ranging from 5 to 18 months. First-line alectinib was used in four patients, with maintenance of efficacy ranging from 5 to 17 months. One patient treated with first-line ceritinib achieved maintenance of efficacy of 26 months, and one patient treated with chemotherapy showed no effect. Three patients were treated with second-line therapy: one treated with crizotinib and one treated with ensartinib achieved maintenance of 5 and 13 months, respectively, and the remaining patient treated with chemotherapy achieved no clinical benefit. Four patients had TP53 mutations, and the duration of first-line treatment maintenance ranged from 5 to 11 months (5, 6, 7, and 11 months, respectively) (13, 14, 19–21).

Ensartinib is a second-generation ALK-TKI. In the phase I/II eXalt2 trial, ensartinib demonstrated a high response rate in patients with ALK fusions resistant to prior crizotinib treatment (22). Use of ensartinib to treat NSCLC exhibiting STRN-ALK fusion has demonstrated good clinical efficacy in patients with crizotinib-resistant relapsed NSCLC (12).Our patient’s lung function was so poor that he could not tolerate surgical treatment; he also had many concomitant diseases and a low PS score.

Considering our patient’s advanced age and poor underlying condition, we used a dose creep of ensartinib and achieved a therapeutic response at the 150 mg dose. COVID-19 subsequently resulted in respiratory failure combined with heart failure, and ensartinib was continued at 200 mg/day with some clinical success. Notably, this is the oldest reported patient with COVID-19 who had STRN-ALK fusion and was treated by first-line ensartinib. Furthermore, the patient showed coalteration of TP53. A previous study reported that TP53 mutations disrupt the tumor suppressor function of the p53 protein and can be detected in almost all types of cancer, including ALK-positive NSCLC, in which TP53 mutations are the most common genomic alteration detected. Patients with this mutation generally have poor progression-free survival (23). In four previously reported cases of STRN-ALK treated with ALK-TKIs, the sustained response time ranged from 5 to 11 months (5, 6, 7, and 11 months, respectively) (13, 14, 19–21). After 13 April 2023, we increased the dose of ensartinib to 225 mg/day in our patient with the goal of achieving a good long-term clinical outcome.

## Conclusion

This case provides valuable clinical evidence of the response to ensartinib as a first-line treatment for patients with NSCLC exhibiting rare STRN-ALK fusions. Our patient had a very poor underlying condition, advanced age, and type 1 respiratory failure and heart decompensation due to COVID-19; nevertheless, he was still able to tolerate treatment with ensartinib. The presence of the TP53 mutation may be associated with poor clinic outcomes.

## Data availability statement

The raw data supporting the conclusions of this article will be made available by the authors without undue reservation.

## Ethics statement

The studies involving humans were approved by Changxing county hospital ethics committee. The studies were conducted in accordance with the local legislation and institutional requirements. Written informed consent for participation in this study was provided by the participants’ legal guardians/next of kin. Written informed consent was obtained from the individual(s), and minor(s)’ legal guardian/next of kin, for the publication of any potentially identifiable images or data included in this article.

## Author contributions

G-QS and Y-ZL have contributed equally to this work and share first authorship (G-QS write and polish this paper, Y-ZL do some check work), WK confirm the pathology and check the figures. G-QH was responsible for the design of the paper, the cost and the confirmed end edition, who can be considered as a corresponding author. All authors contributed to the article and approved the submitted version.
